# Calibrating multiplex serology for *Helicobacter pylori*

**DOI:** 10.1186/s41512-025-00202-x

**Published:** 2025-08-11

**Authors:** Emmanuelle A. Dankwa, Martyn Plummer, Daniel Chapman, Rima Jeske, Julia Butt, Michael Hill, Tim Waterboer, Iona Y. Millwood, Ling Yang, Christiana Kartsonaki

**Affiliations:** 1 Harvard T. H. Chan School of Public Health, Boston, MA USA; 2 Clinical Trial Service Unit & Epidemiological Studies Unit, Nuffield Department of Population Health, University of Oxford, Oxford, OX3 7LF UK; 3 Department of Statistics, University of Warwick, Coventry, UK; 4 Infections and Cancer Epidemiology Division, German Cancer Research Center, Heidelberg, Germany

**Keywords:** *Helicobacter pylori*, Classification algorithms, Multiplex serology, Immunoblot, Prediction, Supervised learning

## Abstract

**Background:**

*Helicobacter pylori* (*H. pylori*) is a bacterium that colonizes the stomach and is a major risk factor for gastric cancer, with an estimated 89% of non-cardia gastric cancer cases worldwide attributable to *H. pylori*. Prospective studies provide reliable evidence for quantifying the association between gastric cancer and *H. pylori*, as they circumvent the risk of a false negative due to possible reduction in antibody levels before cancer development.

**Methods:**

In a large-scale prospective study within the China Kadoorie Biobank, *H. pylori* infection is being analysed as a risk factor for gastric cancer. The presence of infection is typically determined by serological tests. The immunoblot test, although well established, is more labour intensive and uses a larger amount of plasma than the alternative high-throughput multiplex serology test. Immunoblot outputs a binary positive/negative serostatus classification, while multiplex outputs a vector of continuous antigen measurements.

When mapping such multidimensional continuous measurements onto a binary classification, statistical challenges arise in defining classification cut-offs and accounting for the differences in infection evidence provided by different antigens. We discuss these challenges and propose a novel solution to optimize the translation of the continuous measurements from multiplex serology into probabilities of *H. pylori* infection, using classification algorithms (Bayesian additive regressive trees (BART), multidimensional monotone BART, logistic regression, random forest and elastic net). We (i) calibrate and apply classification models to predict probabilities of *H. pylori* infection given multiplex measurements, (ii) compare the predictive performance of the models using immunoblot as reference, (iii) discuss reasons for the differences in predictive performance and (iv) apply the calibrated models to gain insights on the relative strengths of infection evidence provided by the various antigens.

**Results:**

All models showed high discriminative ability with at least 95% area under the curve (AUC) estimates on the training and test data. There was no substantial difference between the performance of models on the training and test data.

**Conclusions:**

Classification algorithms can be used to calibrate the *H. pylori* multiplex serology test to the immunoblot test in the China Kadoorie Biobank. This study furthers our understanding of the applicability of classification algorithms to the context of serologic tests.

**Supplementary Information:**

The online version contains supplementary material available at 10.1186/s41512-025-00202-x.

## Introduction

*Helicobacter pylori* (*H. pylori*) is a bacterium that colonizes the stomach. It causes gastric cancer and gastric mucosa-associated lymphoid tissue (MALT) lymphoma [16] and may be associated with colon cancer [33]. Globally, *H. pylori* causes 810,000 cancer cases annually [22].

Accurate assessment of *H. pylori* infection status has been a major methodological challenge in epidemiological studies of gastric cancer, where infection status is typically evaluated with serologic assays that measure *Ig*G antibodies to *H. pylori* antigens. Advanced atrophic gastritis, a precursor of gastric cancer, can cause a reduction in antibody levels [18] which may lead to a negative test result in a cancer case despite a history of infection with *H. pylori*. Such false-negative findings attenuate the apparent association between *H. pylori* and gastric cancer. For this reason, the most reliable evidence for quantifying the relationship between *H. pylori* and gastric cancer comes from prospective studies with blood collection many years before diagnosis [16]. One such study is the China Kadoorie Biobank.

The China Kadoorie Biobank (CKB) [7] is a prospective study of half a million Chinese adults aged 30–79 years at the time of recruitment (2004–2008) which aims to analyse risk factors associated with many chronic diseases in the Chinese population. Infectious agents in the CKB population are measured by a custom multiplex serology panel that simultaneously measures 43 antigens for 19 pathogens [37] including 12 antigens for *H. pylori* [24].

In the present study, we consider the calibration of multiplex serology results for *H. pylori* in the CKB. The multiplex assay produces median fluorescence intensity (MFI) measurements on 12 *H. pylori* antigens. These 12 continuous measurements must be translated to a single binary positive/negative classification. In previous applications, this has been done by choosing cut-offs for each antigen and then classifying an individual as positive if four or more antigens had MFIs above their respective cut-offs [24]. This is not an optimal use of the test results. The practice of dichotomizing continuous predictors in biomedical research has been criticized on the grounds that it throws away useful information [1, 21, 32]. In addition, the decision rule that classifies an individual as positive if four or more antigens are above their respective cut-offs puts all antigens on equal footing. It is more likely that some antigens provide stronger evidence of *H. pylori* infection than others. In the present study, we investigate an alternative strategy using machine learning methods to optimize the binary classification.

A case-cohort study of *H. pylori* infection and gastric cancer was conducted in the CKB [36]. All participants in the case-cohort study were tested with multiplex serology, and a subsample was additionally tested with immunoblot (Helicoblot 2.1, Genelabs Diagnostics) (Genelabs [11]). This created an opportunity to calibrate multiplex serology against immunoblot, which has shown promising results in previous studies. Immunoblot has shown stronger associations between *H. pylori* and gastric cancer compared to ELISA [27]. Additionally, immunoblot does not discriminate between past and current *H. pylori* infections [34]. This may be an advantage in epidemiological studies where past infections may still constitute an important risk factor for gastric cancer. However, immunoblot is not scalable to large biobank studies as it is slow, labour intensive and requires a relatively large amount of plasma or serum compared to multiplex serology.

Statistically, the calibration problem may be considered a supervised learning problem for which a number of different classification algorithms are available (e.g. logistic regression, random forest, elastic net, Bayesian additive regressive trees (BART) and multidimensional monotone BART). An appealing characteristic of classification algorithms for use in this context is their ability to account for the differences in strength of evidence of infection provided by the various antigens. By design, the output of a classification model is based on predictor (here, antigen) weights which explain the relative influence of a predictor on the outcome (here, *H. pylori* serostatus). Also, classification models output probabilities, which allow for pragmatic cut-off points to be later decided, if necessary. We however provide predictions as probabilities due to the downsides of dichotomization, earlier referenced [1, 21, 32].

The work is structured as follows. In the “Methods” section, we introduce key concepts, the data sets considered, the algorithms compared, the performance metrics employed in evaluating prediction performance and the methods used in evaluating antigen importance. The “Methods” section concludes with an outline of the analysis workflow. In the “Results” section, we present study results and conclude in the “Conclusions” section with a discussion of key results.

## Methods

In this section, we discuss five main themes: (1) the data sets utilized, (2) the classification algorithms compared, (3) antigen importance, (4) performance metrics used in assessing the models’ predictive performance and (5) the overall analysis workflow. All analyses were performed in R (version 4.0.5) [28].

### Data

The data used in this study are quantities from the immunoblot and multiplex serology tests on China Kadoorie Biobank participants. All data were anonymized.

#### Serologic tests

##### Immunoblot

The assay and criteria for determining *H. pylori* seropositivity in the China Kadoorie Biobank, based on immunoblot, have been described in detail elsewhere [36]. The test detects antibodies to seven *H. pylori* antigens, including cytotoxin-associated gene A (CagA), vacuolating cytotoxin A (VacA) and Urease A (UreA) and yields one of three outcomes: (1) *H. pylori* seropositive, (2) *H. pylori* seronegative and (3) ambiguous, suggesting an inconclusive result. For the purposes of this study, we considered ambiguous results to be negative. The accuracy of the immunoblot test used in this study (Helicoblot 2.1) has been evaluated by Monteiro et al. [25], Veijola et al. [34] and Biranjia-Hurdoyal and Seetulsingh-Goorah [3]. Monteiro et al. [25] found that immunoblot correctly classified 51/52 (98%) *H. pylori*-positive patients and 55/64 (86%) *H. pylori*-negative patients using PCR in gastric biopsies as a gold standard. Veijola et al. [34] found correct classification in 27/27 (100%) patients with current *H. pylori* infection and 19/20 (95%) patients with no known infection using histologic stains in gastric biopsies as a gold standard. In addition, they found a positive immunoblot result for 77/84 (92%) patients with past *H. pylori* infection, leading them to conclude that immunoblot does not distinguish past and current infection. Biranjia-Hurdoyal and Seetulsingh-Goorah [3] reported sensitivity of 98.9% and specificity of 70.8% against stool samples with HpStaR as a gold standard.

##### *H. pylori* multiplex serology

Multiplex serology quantifies the level of antibody response to 13 *H. pylori* antigens. The test procedure is described elsewhere [24]. Briefly, recombinantly expressed *H. pylori* proteins were loaded separately onto fluorescence-labelled *beads*. After the antigen-loaded beads had been mixed, they were incubated with 1:1000 diluted sera. A *Luminex 200 analyser* then identified the bead type and consequently the bound antigen and simultaneously quantified the fluorescent signal corresponding to the amount of antibodies bound to the antigen-loaded beads. The output is given as the median fluorescence intensity (MFI) of at least 100 beads.

The proteins considered in the multiplex assay used in the China Kadoorie Biobank are as follows: CagA, VacA, chaperonin GroEL (GroEL), HP1564 (Omp), neuraminyllactose-binding hemagglutinin homolog (HpaA), hydantoin utilization protein (HyuA-C), cinnamyl alcohol dehydrogenase ELI3-2 (Cad), urease alpha subunit (UreA), neutrophil-activating protein (NapA), conserved hypothetical secreted protein (HcpC), catalase, and a hypothetical protein (HP0305). These antigens were selected [24] based on known immunogenicity (UreA, GroEl, NapA, CagA, VacA, HpaA) or suggested serological association with gastric disease (Cad, HyuA, Omp, HcpC, HP0305). Michel et al. [24] reported a sensitivity of 89% and a specificity of 82% against commercial assays (screening ELISA with Western blot confirmation).

We note that the two assays measure different antigens, with some overlap. This raises the question of whether one assay can be used to calibrate the other. We proceed on the basis that the underlying true state we are trying to assess is binary presence or absence of *H. pylori* infection. The individual antigens are indirect indicators of this underlying dichotomy.

#### Training and case data

The case-cohort study in the China Kadoorie Biobank excluded subjects who satisfied any of the following criteria: (i) Unavailable blood samples, (ii) cancer diagnosis prior to baseline, (iii) cancer diagnosis within the first 2 years of follow-up, (iv) death or loss to follow-up within the first 2 years of follow-up and (v) inconsistencies in data [36]. Here, we refer to the cohort of subjects which remained after the application of this exclusion criteria as the *eligible cohort*. In keeping with the design of a case-cohort study [20], a random subset of 2000 participants, stratified by age and sex, was selected from among the eligible cohort to form the *subcohort*.

Both immunoblot and multiplex serology were conducted on all cases of gastric cancer in the eligible cohort as well as on a fraction of subjects in the subcohort. The data from the serologic tests on this fraction of the subcohort (*N* = 498) was used for model training. Immunoblot testing was only feasible on a small number of participants due to its high cost. We considered the subcohort data too small to split into training and validation sets and therefore relied on cross-validation to assess the prediction error in the subcohort data as described below. The predictive accuracy was also tested on the gastric cancer cases (*N* = 922). Note however that the cases are expected to have a much higher prevalence of *H. pylori* than the subcohort data due to the known association of *H. pylori* with gastric cancer risk.

Antibody reactivities, by multiplex serology, were used as predictors (input variables) for *H. pylori* seropositivity by immunoblot (outcome variable). Thus, each data point used in model training and evaluation is of the form of Eq. (1):

1 $$\left({y}_{j},{x}_{1j},\dots {x}_{kj}\right), j = 1,\dots N,$$

where ${y}_{j}$ is the *H. pylori* infection status of subject *j*, taking on the value 0 if subject *j* is *H. pylori* seronegative and the value 1 if participant *j* is *H. pylori* seropositive. The value ${x}_{ij}$ represents antibody reactivity corresponding to antigen $i (i = 1,...,12)$ for participant *j* measured by multiplex serology. Prior to analysis, all predictor variables were converted to the log scale to control for the large variation observed within variables and standardized to have sample mean 0 and variance 1 across individuals.

### Algorithms

Here, we give brief theoretical overviews of all algorithms considered. Our main focus is on algorithms based on decision trees as these are the natural generalization of the classification algorithm of Michel et al. [24]. Recall that Michel et al. [24] classified a multiplex test result as positive if four or more antigens were above their respective cutoff levels. A decision tree is an algorithm based on recursively splitting the data into two groups based on one of the predictor variables ${x}_{i}$ according to whether it is above or below a given threshold. This can be represented as a tree in which nodes represent splitting rules and branches represent different paths taken by different values of the predictor variables ${\varvec{x}}$. Given predictor variables ${{\varvec{x}}}_{{\varvec{i}}}$ for individual $i$, we follow a path from the root node to one of the terminal nodes, which determines the prediction for the outcome variable ${{\varvec{Y}}}_{i}$.

An individual decision tree is highly sensitive to changes in the training data and has large generalization error. Here, we use classifiers based on ensembles of trees, which are combined to produce a strong learner. We consider the random forest, Bayesian additive regression trees (BART) and multidimensional monotone BART (mBART) algorithms. We also consider logistic regression, with and without regularization, as this is one of the most commonly used algorithms in biostatistics. We distinguish between an algorithm and a model: an algorithm is a distinct procedure or method, whereas the model is the result of implementing the algorithm using data. Details of all algorithms are in the supplementary material.

### Antigen importance

We examined how insights on the influence of antigens on *H. pylori* serostatus may be gained from the models developed here. We were interested in identifying the important antigens for *H. pylori* serostatus prediction according to each model and studying the extent to which different models agree on their importance.

For logistic regression, the influence of a predictor on an outcome is measured by the statistical significance and size of the coefficient estimate of the predictor variable [26]. In particular, predictors with large and statistically significant coefficients are considered highly influential, whereas predictors with small coefficients are considered less important for explaining the outcome.

In a random forest, the importance of a predictor may be estimated as a measure of the decrease in prediction accuracy when that predictor is excluded from the model [5]. This is based on the principle that the exclusion of a predictor which highly influences the outcome is expected to result in a substantial decrease in prediction accuracy. Details are in the supplementary material.

For BART, we assessed the importance of a predictor ${{\varvec{x}}}_{i}$ by estimating the proportion of times it is used in splitting a node; this is termed the *inclusion proportion* of ${{\varvec{x}}}_{i}$ [17]. The principle underlying this method is the fact that influential variables (i.e., antigens) are more likely to be employed in node splitting due to their high relatedness with the outcome. Hence, predictors with higher inclusion proportions are considered more important for explaining the outcome.

Using BART antigen importance results as the baseline, we studied the extent to which the three models agree on variable importance, using the following steps.

1. For each model as follows:
  - Compute antigen importance scores and rank predictors in order of decreasing score.
  - To each predictor, assign a rank value based on the predictor’s position in the ordering obtained at 1a. Assign ranks 1, 2 and 3 to the top four, middle four, and last four predictors, respectively.
2. For each predictor, calculate the following:
  - The absolute difference between its rank according to the logistic model and its rank according to BART
  - The absolute difference between its rank according to random forest and its rank according to BART

For a given predictor, the possible values for the absolute difference in ranking between any two of the models being compared are 0, 1 and 2, which we interpret as “perfect agreement”, “moderate agreement” and “poor agreement”, respectively. The lower the absolute rank difference, the higher the agreement on antigen importance between the two models.

The elastic net and mBART models were not considered in the antigen importance analysis. The coefficients from the elastic net model are not interpretable as measures of antigen importance in the presence of interaction terms. For mBART, as far as we know, the implementation available from the authors does not allow for the estimation of variable importance. At the time of writing, we are not aware of any implementations of mBART which permit this analysis.

### Performance metrics

All of our models create probabilistic predictions. Instead of producing a binary prediction ${Y}_{i}=1$ or ${Y}_{i}=0$ for the immunoblot serostatus, each model produces a probability estimate ${\widehat{p}}_{i}$ that ${Y}_{i}=1$ based on the model fitted to the training data. To compare the performance of the five models, we employed three performance metrics: the Brier score, the logarithmic score and the area under the receiver-operator characteristic curve. As each of these performance metrics has its downsides, we chose to use all three in order to obtain a balanced assessment of the methods considered.

For *N* observations in the testing set, let ${\varvec{p}}\boldsymbol{ }= \{{\widehat{p}}_{1},{\widehat{p}}_{2},...,{\widehat{p}}_{N}\}$ be a vector of probabilistic predictions by a classification model and let ${\varvec{Y}}\boldsymbol{ }= \{{Y}_{1},{Y}_{2},...,{Y}_{N}\}$ be the binary outcome representing *H. pylori* serosatus according to immunoblot.

#### Brier score

The Brier score [6] is the average of the squared prediction errors and is given by the following:

2 $$\frac{1}{N}\sum\limits_{i=1}^{N}{\left({\widehat{p}}_{i}-{Y}_{i}\right)}^{2} .$$

The Brier score is negatively oriented (lower scores are better) and assumes values in the interval [0*,*1]. It is a *strictly proper scoring rule* [35], i.e., it is maximized if and only if the predicted probability distribution exactly matches the true (observed) distribution [12]. Here, we use the Brier score as the primary evaluation metric. That is, for a given problem, a prediction with the largest (resp. smallest) probability for an individual who is indeed *H. pylori* seropositive will be rewarded (resp. penalized) the most. A downside of the Brier score is its low sensitivity to small changes in extreme predictions [2].

#### Logarithmic score

The logarithmic score [13] (also “ignorance” score or log loss) is a measure of the cross-entropy (Kullback–Leibler loss) between the prediction distribution and the true distribution of events [2, 29]. The logarithmic score is given by the following:

3 $$-\frac{1}{N}\sum\limits_{i=1}^{N}\left[{Y}_{i}\text{log}{\widehat{p}}_{i}+\left(1-{Y}_{i}\right)\text{log}\left(1-{\widehat{p}}_{i}\right)\right] .$$

The logarithmic score takes on values in the interval $[0, \infty ]$. Like the Brier score, it is negatively oriented and is a proper scoring rule. The logarithmic score has been criticized for its hypersensitivity to negligible differences between small predicted probabilities and for its low sensitivity, under some conditions, to changes in the relationship between the observed and predicted distributions [31].

#### Area under the curve

The area under the curve (AUC) refers to the area under the receiver operating characteristic (ROC) curve. The ROC curve is a two-dimensional plot of the true-positive rate (sensitivity) against the false-positive rate (specificity) for a given classifier [23]. The AUC summarizes the discriminative ability of a classifier [9, 15]. In the current context, the AUC is equivalent to the probability that the classifier ranks a random *H. pylori* seropositive observation higher than a random *H. pylori* seronegative observation. The AUC takes values in the interval [0,1] for a ROC plot with unit area. The closer the AUC is to 1, the better the discriminative ability of the classifier. A classifier with AUC equal to 0.5 possesses the same discriminative ability as a random classifier.

The AUC is unaffected by arbitrary classification threshold choices as it is an average across a number of thresholds [9]. However, the AUC is incoherent in its treatment of misclassification costs across different classifiers and can be misleading when ROC curves cross [14] or when there are severe class imbalances [30].

### Analysis workflow

To compare the performance of the five classification methods and to gain insights into the influence of individual antigens on *H. pylori* status, we used an analysis workflow with three stages summarized as follows: (1) Compare predictive performance of models on training data, (2) compare predictive performance of models on case data and (3) study antigen importance.

#### Stage 1: Compare predictive performance of models on training data

We trained all models via tenfold cross-validation (CV) and computed performance scores at each CV iteration. The training data (*N* = 498) was divided into 10 approximately equally sized parts (folds) such that all folds had approximately the same distribution of *H. pylori* positive and negative individuals as the training data. This was to ensure that models were trained on samples that were representative of the entire training data. The same folds were used for all models to ensure fairness.

Except for logistic regression which has no hyperparameters, all models used fivefold nested CV for selection of the best hyperparameter values at each iteration of the tenfold CV. This ensured better prediction accuracy and greater flexibility than would have achieved in a no-hyperparameter-tuning case, since models could adapt to the different training folds. In the BART and elastic net models, hyperparameters were selected by minimizing the root-mean-square error [17] and the misclassification error, respectively [10]. For random forest and mBART, hyperparameters were chosen to maximize the classification accuracy [19].

For each model, this analysis yielded 10 estimates each of the Brier score, logarithmic score and AUC. The mean and standard deviations of these estimates across folds provided a measure of comparison of predictive performance.

#### Stage 2: Compare predictive performance of algorithms on case data

The predictive performance of all algorithms was tested on the case data (*N* = 922). The algorithms were first trained via tenfold CV on the training data to allow the selection of the best hyperparameter values. Unlike at stage 1, the folds used in training at this stage were not fixed, as comparison on the training data was not the aim here. For each model, we used the best hyperparameter values from the tenfold CV to predict *H. pylori* serostatus in the case data. Given the predictions, we computed performance estimates for each model.

#### Stage 3: Study antigen importance

Using the best logistic regression, random forest and BART models on the training data, we computed antigen importance measures for each antigen and compared results across models.

For logistic regression, the absolute standardized regression coefficients and corresponding standard errors were considered as antigen importance estimates. The level of statistical significance for all coefficient estimates was *α* = 0*.*05. For random forest, the decrease in accuracy for each predictor in the random forest model was computed and averaged over 500 trees using the steps outlined in the “Antigen importance” section. For BART, we computed the inclusion proportions for all predictors in 100 replications of the model with *m* = 20 trees in each replication. The small number of trees was to ensure competition among predictors for inclusion in splitting rules, such that highly influential predictors (which yield greater accuracy when used for mode splitting) will tend to be preferred and hence have higher inclusion proportions than unimportant predictors [4, 8]. For each predictor, we computed the average inclusion proportion and the standard deviation of inclusion proportions across the 100 model replications.

To determine the extent of agreement between models on antigen importance, we computed the absolute differences in antigen rankings between the BART (baseline), random forest and logistic regression models, using the ranking method described in the “Antigen importance” section.

## Results

The seroprevalence of *H. pylori* infection in the training and case data sets was 75.7% and 93.5%, respectively. We report training, validation and antigen importance results according to the analysis workflow.

### Stage 1: Performance of algorithms on training data

Estimates of the predictive performance of the final calibrated models — that is, the models yielding the best performance on the training data — are shown in Table 1. We present the mean CV scores across the 10 folds $\pm$ one standard deviation to represent the variation in scores across folds. The hyperparameter values of the final calibrated models are reported in Tables S1, S2 and S3 and Figure S1 of the supplementary material, along with the predictive accuracy scores (with standard deviations) of all other hyperparameter values considered in the calibration process.

**Table 1:** Predictive performance of the logistic regression, elastic net, random forest, Bayesian additive regression trees (BART) and multidimensional monotone BART (mBART) models on the training data (*N* = 498). The table displays mean $\pm$ one standard deviation of tenfold CV scores. Performance metrics considered were the Brier score, logarithmic score and the area under the receiver operating characteristic curve (AUC). The best score for each metric is in bold

	Performance metric		
**Algorithm**	**Brier score**	**Logarithmic score**	**AUC**
Logistic regression	0.0801±0.02	0.2535±0.07	0.9513±0.03
Elastic net	0.0765±0.02	**0.2441±0.04**	0.9523±0.02
Random forest	0.0767±0.03	0.2560±0.08	0.9581±0.03
BART	**0.0746±0.02**	0.2454±0.06	**0.9583±0.03**
mBART	0.0774±0.01	0.2552±0.04	0.9559±0.02

Overall, BART performed best according to the Brier score and AUC metrics, while elastic net had the best logarithmic score. Using the Brier score, the ranking of models in order of decreasing classification performance on the training data is as follows: BART, elastic net, random forest, logistic regression and mBART.

### Stage 2: Performance of algorithms on case data

Estimates of the predictive performance of the final calibrated models on the case data are provided in Table 2. BART had the best performance according to the Brier score (0.0745) and the logarithmic score (0.2313), while elastic net had the highest AUC (0.9716). Logistic regression had the lowest performance on both the Brier and logarithmic scores, although on the AUC it performed better than random forest and BART. There are no error estimates associated with the performance estimates on the case data because, unlike the training data, performance estimates were not computed on different data folds.

**Table 2:** Performance scores of the logistic regression, elastic net, random forest, Bayesian additive regression trees (BART) and multidimensional monotone BART (mBART) models on the case (test) data (*N* = 922). For all algorithms except logistic regression, the predictive model was selected via tenfold cross-validation on the training data. Performance metrics considered were the Brier score, logarithmic score and the area under the receiver operating characteristic curve (AUC). The best score for each metric is in bold

	Performance metric		
**Algorithm**	**Brier score**	**Logarithmic score**	**AUC**
Logistic regression	0.0876	0.2991	0.9695
Elastic net	0.0804	0.2693	**0.9716**
Random forest	0.0776	0.2522	0.9499
BART	**0.0745**	**0.2313**	0.9558
mBART	0.0752	0.2372	0.9573

### Stage 3: Antigen importance

**Fig. 1:**
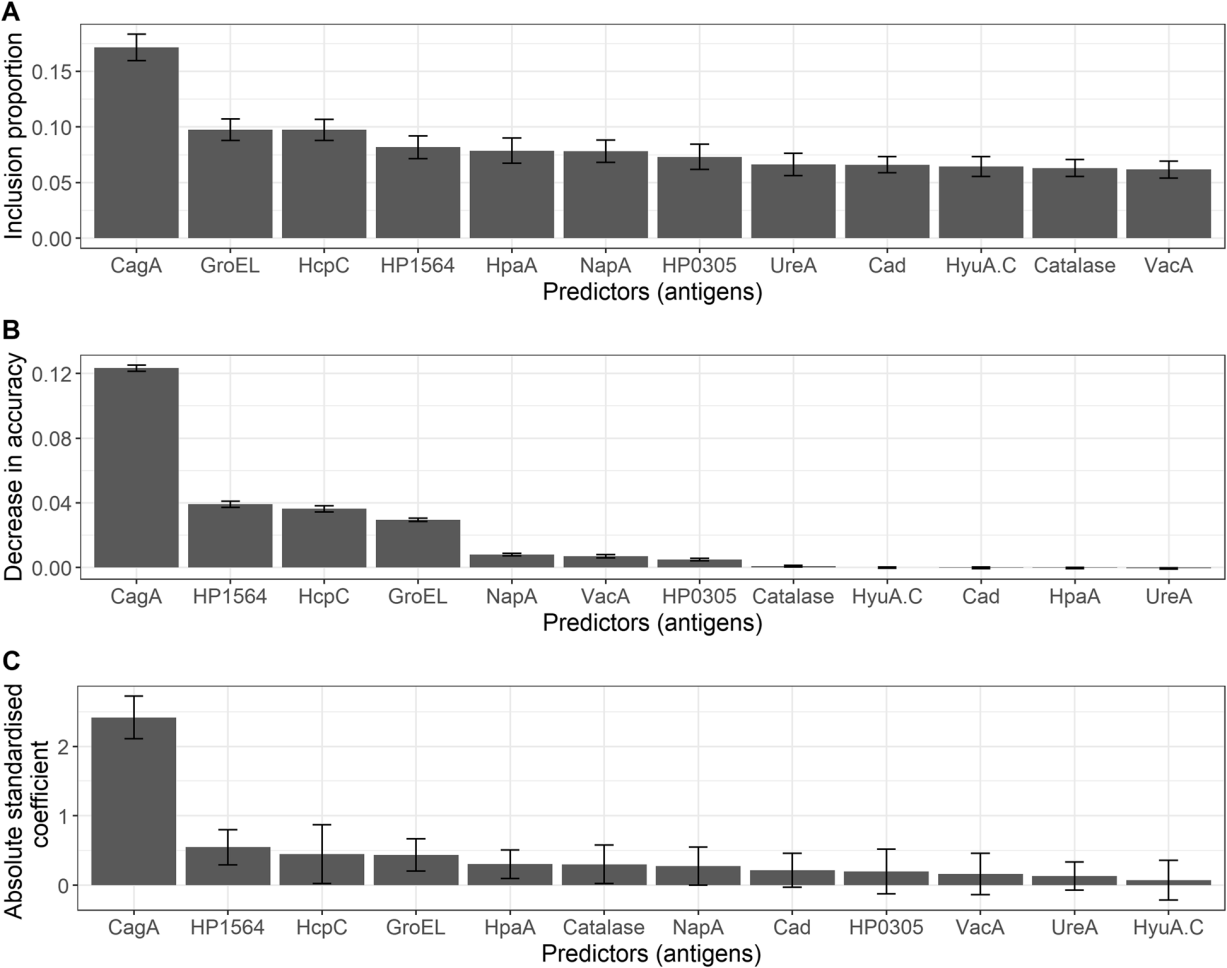
Antigen importance measures based on the Bayesian additive regression trees (BART), random forest and logistic regression models trained on the complete analysis cohort data. **A** Mean inclusion proportions for predictors, averaged across 100 replications of a BART model, each with 20 trees. Error bars represent standard deviations of estimates. **B** Mean decrease in accuracy corresponding to each predictor, averaged over 500 trees in the random forest. Error bars represent standard deviations of estimates. **C** Absolute standardized regression coefficients for the logistic regression model. Error bars represent the standard errors of coefficient estimates

Antigen importance scores with corresponding variance estimates for all antigens are shown for BART (Fig. 1A), random forest (Fig. 1B) and logistic regression (Fig. 1C) trained on the complete training data.

**Fig. 2:**
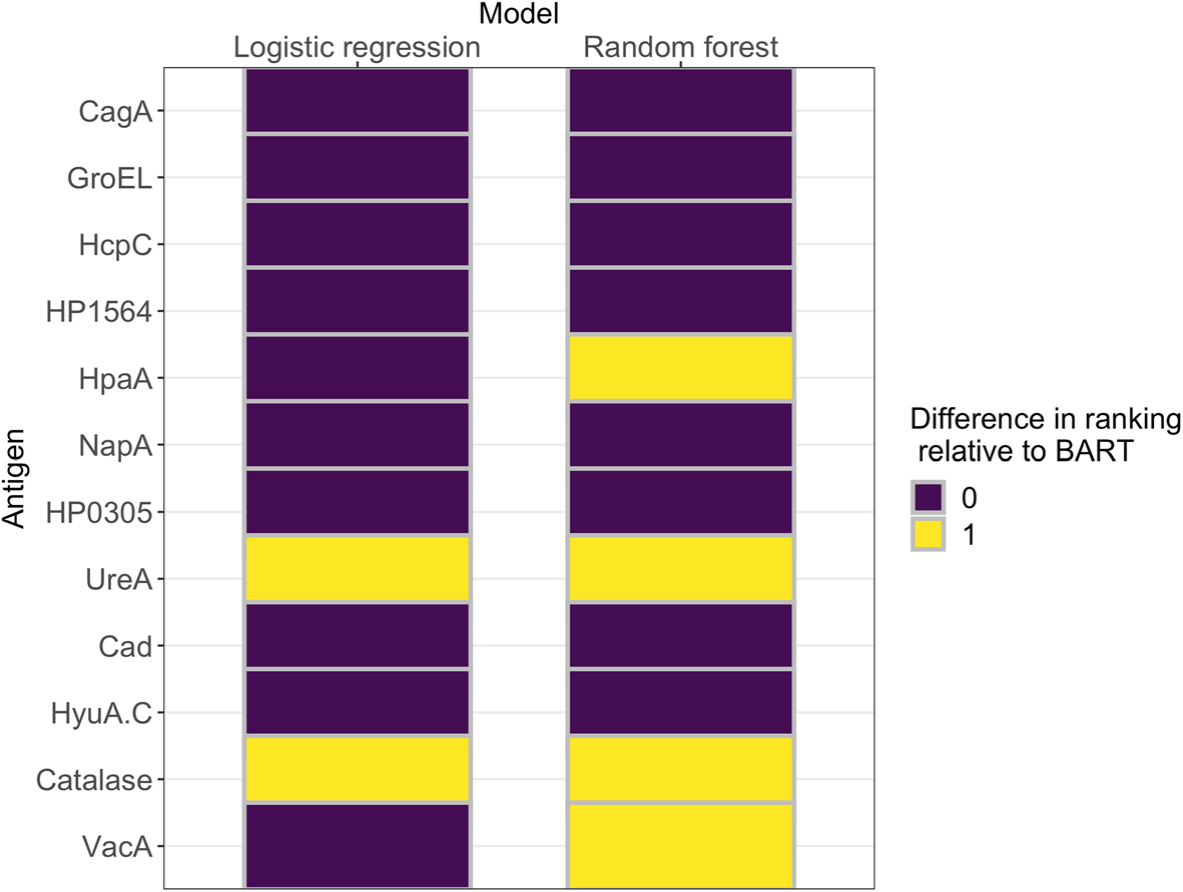
Absolute differences in antigen importance ranking by logistic regression and random forest, relative to Bayesian additive regression trees (BART) rankings. Lower absolute differences indicate higher rank agreement between methods: a rank of “0” (purple) indicates no difference with BART rankings, and a rank of “1” (yellow) indicates a difference in one rank level between the model's ranking and BART's rankings

The absolute differences in antigen importance rankings between BART (baseline), random forest and logistic regression are shown in Fig. 2. There was perfect agreement in the ranking of the four most influential antigens, as all models ranked CagA, HcpC, GroEL and Omp in the top 4. Also, the three models agreed on the ranking of two of the least influential antigens: UreA and HyuA.C. The BART rankings agreed with both random forest and logistic regression rankings for 8 of 12 antigens ($\approx$ 67%), namely CagA, HcpC, GroEL, Omp, HP0305, NapA, Cad and HyuA.C. The poorest ranking agreement between BART and the random forest was observed with UreA, Catalase, VacA and HpaA. In all three models, CagA had the highest influence on *H. pylori* serostatus.

## Conclusions

We have compared the performance of five classification algorithms — logistic regression, elastic net, random forest, BART and mBART — on *H. pylori* serologic data in the China Kadoorie Biobank. All trained models showed high discriminative ability, as all AUC estimates on the training and case data were at least 95% (Tables 1 and 2). The best discriminative ability (*AUC* $=9$7%) was exhibited by the mBART model on the case data. We did not observe a substantial difference between the performance of models on the training data and their performance on the case data.

By the Brier score, BART had the best predictive performance on both the training and case data sets, although the differences between scores of the different models were small (Tables 1 and 2). The superior performance of the BART, mBART and random forest models in comparison to logistic regression is likely due to their ability to automatically account for interactions between predictors. Although logistic regression models can include interaction terms, we considered that without regularization, this would introduce instability in the model. Interaction effects were included in the elastic net model which showed a slightly better performance than logistic regression but a generally lower performance compared to the tree-based methods (BART, mBART and random forest). The automatic incorporation of predictor interactions is an appealing property of tree-based methods. Although not utilized here, the Bayesian methods (BART and mBART) also have the advantage of adapting to changes in prior information on the relationships between predictors [8].

The BART, random forest and logistic regression models showed high levels of agreement in antigen importance ranking, with an agreement rate of 67% on the 12 antigens considered (Fig. 2). These models agreed on CagA as the most influential antigen for the determination of *H. pylori* serostatus and on Cad and HyuA.C as among the least influential. The result for CagA is consistent with findings from previous studies. An earlier study on data from the China Kadoorie Biobank showed CagA as having the strongest associations with the risk of gastric cancer [36]. Additionally, Michel et al. [24] found CagA as the most immunogenic antigen among 17 *H. pylori* proteins and observed a high agreement for CagA (kappa: 0.77; 95% confidence interval (*CI*): 0.69–0.86) between immunoblot and multiplex serology results on 227 reference sera. Both assays used in this study test for antibodies to CagA. In contrast, the VacA and UreA proteins that are also common to both assays were not highly ranked by antigen importance.

Contrary to our expectations, the imposition of monotonicity constraints on the BART framework did not lead to improvements in predictive ability. It is not yet clear why mBART did not outperform BART, although the current prediction problem satisfies the monotonicity condition that higher antibody reactivity always implies increased probability of infection.

The study could be improved in a number of ways. First, statistical tests could be performed to determine which differences in performance scores between algorithms were due to random chance. Given the small differences observed between scores, such insights are needed to further elucidate the differences in predictive ability between these algorithms. Second, scores on the case data could be reported separately by *H. pylori* serostatus to help determine whether some algorithms are particularly suited for prediction in specific subgroups. Finally, the study could include an analysis of algorithm runtimes vis-à-vis their prediction accuracy to further our understanding of accuracy–speed trade-offs involved in the application of these algorithms, especially on large data sets as those held in biobanks. As this study is a calibration of a diagnostic assay, we did not consider other potential predictors that are not considered in the diagnostic procedure, for example demographic variables.

While the calibrated models developed here could be used to classify serostatus for all participants in the China Kadoorie Biobank, they might fail to generalize outside of the study population. Obtaining more generalizable models would require a repeat of the calibration exercise in a suitably chosen random subsample of the new study population, as we would expect the serological profile of the population to be different. Two key features of the China Kadoorie Biobank population are relevant to the generalizability of these results. Firstly, this is a high-prevalence population with 75.7% *H. pylori* infection in the controls. Under these circumstances, the accuracy of any serological test depends on its ability to identify uninfected individuals, i.e. its specificity. In a low-prevalence population, the balance between sensitivity and specificity in terms of diagnostic accuracy will be different. Secondly, a high proportion of infections are CagA positive. Antibodies to CagA have been shown to persist after *H. pylori* eradication and are therefore considered a useful marker of lifetime exposure [34]. Both immunoblot and multiplex assays detect antibodies to CagA, which was the most important variable in the calibration of multiplex serology to immunoblot. It is not clear whether equally good calibration is attainable in a population infected by CagA-negative strains. In conclusion, we have demonstrated how classification algorithms can be used to calibrate the *H. pylori* multiplex serology test to the immunoblot test in the China Kadoorie Biobank. This study furthers our understanding of the applicability of classification algorithms to the context of serologic tests.

## Supplementary Information

Additional file 1: Table S1: Average out-of-bagroot mean square error for different hyperparameter combinations for the BART algorithm on the training data. Scores are displayed for all hyperparameter combinations in order of increasing RMSE. Averages are over validation folds within a tenfold cross-validation scheme. The hyperparameters $m$ and $k$ represent the number of trees and the amount of prior information used in the model, respectively. The best hyperparameter combination, $\{k=2, m=200\}$, was used for prediction among cases. Table S2: Average accuracy scores and standard deviations (SDs) for different hyperparameter combinations for the mBART algorithm on the training data**.** Averages and SDs are over validation folds within a tenfold cross-validation scheme. Scores are displayed for all hyperparameter combinations in order of decreasing accuracy. The hyperparameters $m$ and $k$ represent the number of trees and the amount of prior information used in the model, respectively. The best hyperparameter combination (that which yielded the optimal score across the 10 folds) was used for prediction in the case data. Due to rounding, the accuracy scores of the combinations $\left\{k=2, m=200\right\}$ and $\{k=3, m=200\}$ appear equal; however, the second set has a higher average score and a lower SD. Table S3: Average accuracy scores and standard deviations (SDs) for different hyperparameter combinations for the random forest algorithm on the training data. Averages and SDs are over validation folds within a tenfold cross-validation scheme. Scores are displayed for all possible hyperparameter values in order of decreasing accuracy. The hyperparameter ${m}_{try}$ represents the number of variables used for node splitting. The best hyperparameter combination (that which yielded the optimal score across the 10 folds) $\{{m}_{try}=6\}$, was used for prediction in the case data. Figure S1: Misclassification error for different values of $\lambda$ in the elastic net (EN) algorithm on the training data within a tenfold cross-validation (CV) scheme. Red dots and grey bars represent the average misclassification error and standard deviation of misclassification errors, respectively, across the 10 CV folds for each value of $\lambda$. The dashed vertical lines are plotted at the value of $\lambda$ with the minimum average CV error (first dashed line from the left) and at the largest value of $\lambda$ such that the error is within one standard error of the minimum (second dashed line from the left). The numbers displayed at the top of the plot represent the number of non-zero coefficient estimates at the corresponding values of $\lambda$. The value of $\lambda$ with the minimum average CV error $(log(\lambda)= -3.93)$ was used within the EN model for prediction on the case data. The value of $\alpha$ (EN mixing parameter) was fixed at 0.90 to allow more weight towards a lasso penalty ($\alpha$ = 1) while still allowing for a ridge penalty ($\alpha=0$) to control multicollinearity effects.

## Data Availability

No datasets were generated or analysed during the current study.

## Abbreviations

AUC: Area under the curve
BART: Bayesian additive regression trees
Cad: Cinnamyl alcohol dehydrogenase ELI3-2
CagA: Cytotoxin-associated gene A
CV: Cross-validation
ELISA: Enzyme-linked immunosorbent assay
GroEL: Chaperonin GroEL
HcpC: Conserved hypothetical secreted protein
*H. pylori*: *Helicobacter pylori*
HpaA: Neuraminyllactose-binding hemagglutinin homolog
HyuA-C: Hydantoin utilization protein
mBART: Multidimensional monotone Bayesian additive regression trees
NapA: Neutrophil-activating protein
UreA: Urease alpha subunit
