# Supplementary material for: Calibrating multiplex serology for Helicobacter pylori
Source: Diagn Progn Res. 2025 Aug 11;9:17. doi: 10.1186/s41512-025-00202-x (PMC12337413; doi:10.1186/s41512-025-00202-x)
Supplement: Supplementary file 1 — Additional file 1: Table S1: Average out-of-bagroot mean square error for different hyperparameter combinations for the BART algorithm on the training data. Scores are displayed for all hyperparameter combinations in order of increasing RMSE. Averages are over validation folds within a tenfold cross-validation scheme. The hyperparameters $m$m and $k$k represent the number of trees and the amount of prior information used in the model, respectively. The best hyperparameter combination, $\{k=2, m=200\}${k=2,m=200}, was used for prediction among cases. Table S2: Average accuracy scores and standard deviations (SDs) for different hyperparameter combinations for the mBART algorithm on the training data. Averages and SDs are over validation folds within a tenfold cross-validation scheme. Scores are displayed for all hyperparameter combinations in order of decreasing accuracy. The hyperparameters $m$m and [file 41512_2025_202_MOESM1_ESM.docx]

**Supplementary Material for “Calibrating multiplex serology for *Helicobacter pylori”***

Emmanuelle A. Dankwa^1,2^, Martyn Plummer^3^, Daniel Chapman^2^, Rima Jeske^4^, Julia Butt^4^, Michael Hill^2^, Tim Waterboer^4^, Iona Y. Millwood^2^, Ling Yang^2^, and Christiana Kartsonaki^2,*^

1. Harvard T. H. Chan School of Public Health, Boston, Massachusetts, USA
2. Clinical Trial Service Unit & Epidemiological Studies Unit, Nuffield Department of Population Health, University of Oxford, Oxford, United Kingdom
3. Department of Statistics, University of Warwick, Coventry, United Kingdom

^4^ Infections and Cancer Epidemiology Division, German Cancer Research Center, Heidelberg, Germany

*Corresponding author: Clinical Trial Service Unit & Epidemiological Studies Unit, Nuffield Department of Population Health, University of Oxford, Old Road Campus, Roosevelt Drive, Oxford OX3 7LF, Oxford, United Kingdom. [christiana.kartsonaki@dph.ox.ac.uk](mailto:christiana.kartsonaki@dph.ox.ac.uk)

Table of Contents

[Algorithm details 3](#_Toc200641541)

[1 Logistic regression 3](#_Toc200641542)

[2. Elastic net 3](#_Toc200641543)

[3. Random forest 4](#_Toc200641544)

[4. Bayesian Additive Regression Trees (BART) 5](#_Toc200641545)

[Prior Specification 5](#_Toc200641546)

[Posterior Distribution 6](#_Toc200641547)

[Implementation in R 7](#_Toc200641548)

[5. Multidimensional Monotone BART (mBART) 7](#_Toc200641549)

[Prior Specification 7](#_Toc200641550)

[Posterior Distribution 7](#_Toc200641551)

[Implementation in R 7](#_Toc200641552)

[Random forest: predictive performance 8](#_Toc200641553)

[Cross-validation results 9](#_Toc200641554)

[References 13](#_Toc200641555)

# Algorithm details

## 1 Logistic regression

Logistic regression (LR) (Berkson, 1944; Cox, 1958) models the relationship between a categorical outcome variable and one or more predictor variables. We use a multivariable binary logistic model,

| $\log\left( \frac{p_{j}}{1-p_{j}} \right) = \beta_{0} + \beta_{1}X_{1j} + \beta_{2}X_{2j} + \cdot\cdot\cdot+ \beta_{k}X_{kj},$ | (1) |
| --- | --- |

where $p_{j}$ is the probability that $Y_{j}= 1$ and $\boldsymbol{\beta} = \left\{ \beta_{o}, \beta_{1},\ldots,\beta_{k} \right\}$ is the vector of regression parameters, which are estimated by maximizing the log likelihood function:

| $l\left( \boldsymbol{\beta} \right)= \sum_{j=1}^{N} \left[ y_{j}\log\left( p_{j} \right)+\left( 1-y_{j} \right)\log\left( 1-p_{j} \right) \right].$ | (2) |
| --- | --- |

For all analyses involving logistic regression, we used the glm function available in the **stats** package in R (R Core Team, 2021).

## 2***. Elastic net***

Elastic net (Zou and Hastie, 2005) is an extension of regression modelling in which the parameters are estimated by maximizing a penalized log likelihood:

| $l\left( \boldsymbol{\beta} \right)-\lambda\left( \alpha\left\Vert\boldsymbol{\beta} \right\Vert_{1}+\left( 1-\alpha\right)\left\Vert\boldsymbol{\beta} \right\Vert_{2} \right),$ | (3) |
| --- | --- |
|  |  |
|  |  |

where $\left\| \boldsymbol{\beta} \right\|_{1}=\sum_{i=1}^{k} \left| \beta_{i} \right|$ is the *L*_1_ norm (or Manhattan distance) and $\left\| \boldsymbol{\beta} \right\|_{2}=\sum_{i=1}^{k} \beta_{i}^{2}$ is the *L*_2_ norm (or Euclidean distance), both ignoring the intercept parameter β_0._ The *L*_2_ penalty shrinks the parameter estimates towards zero. This increases predictive accuracy and improves numerical stability when there are highly correlated predictor variables (Hilt and Seegrist, 1977). The *L*_1_ penalty can shrink elements of the parameter vector **β** to zero, completely removing the influence of the corresponding predictor variables. This improves numerical stability when there are a large number of potential predictor variables in the model(Friedman et al., 2010).

We took advantage of the increased stability of ridge regression to extend the model from equation (3) to

| $\log\left( \frac{p_{j}}{1-p_{j}} \right) = \beta_{0} + \beta_{1}X_{1j} + \beta_{2}X_{2j} + \cdot\cdot\cdot+ \beta_{k}X_{kj},$ | (4) |
| --- | --- |

where the $\beta_{il}$ terms represent interaction effects between the different antigens. With k=12 antibody reactivity values available, the addition of all 2-way interactions adds 66 parameters to the model. This extension is not feasible with the unpenalized logistic regression model as the additional parameters greatly increase the numerical instability and generalization error.

Compared with logistic regression, elastic net introduces two new hyper-parameters: $\lambda\geq0$ controls the overall size of the penalty term and $\alpha\in[0,1]$ controls the balance between the two penalties where $\alpha= 0$yields ridge regression (Hilt and Seegrist, 1977) and $\alpha= 1$ yields the lasso (Tibshirani, 1996). We chose *α* = 0.90 to emphasize the lasso penalty. The hyper-parameter $\lambda$ was chosen by nested cross-validation as described in Figure S1 (Supplementary Material).

Analyses with the elastic net were performed with the **glmnet** package (version 4.1-1) (Friedman et al., 2010).

## 3. Random forest

A random forest (RF) (Breiman, 2001) is a model comprising an ensemble of classification trees. Each tree in the random forest is grown by repeatedly *splitting* (partitioning) its terminal *nodes* (ends) into more homogenous daughter nodes (Ishwaran, 2015) on the basis of whether one of the predictor variables $x_{i}$ is above or below a given cutpoint. Each terminal node is associated with a binary classification in the set {0,1} which determines the prediction for the outcome variable $Y$. The output of the random forest is a predicted probability $\hat{p}$ calculated from the average of the binary prediction over all trees in the ensemble.

The predictive accuracy of the random forest model depends on sufficiently high accuracy of the individual classification trees and low correlation between trees. Random forest uses two techniques to control the correlation between trees and the prediction variance,: *bootstrap aggregating* (bagging) (Breiman, 1996) and randomization of the *node-splitting variables,* which determine how nodes are split. Bagging involves drawing bootstrap samples (Hastie et al., 2009) – random samples drawn with replacement – from the original training set, training a different tree classifier on each of these samples and then aggregating predictions over all trees to obtain the model prediction. This aggregation serves as a variance reduction technique (Boehmke and Greenwell, 2019; Ishwaran, 2015). Bagging also enables the internal estimation of prediction error (Breiman, 1996). Each bootstrap sample leaves a fraction of out-of-bag samples that were not used to train the tree and which can be used to estimate its prediction error.

At each node, the random forest randomly selects a random subset of input variables for node splitting. The number of variables for node splitting $m_{\mathrm{try}},$ is often treated as a hyperparameter as it strongly affects predictive accuracy (Boehmke and Greenwell, 2019). By incorporating two sources of randomness, namely randomization of node-splitting variables and bootstrapping, the random forest controls correlation between trees (Boehmke and Greenwell, 2019; Ishwaran, 2015). A useful attribute of the random forest is that it does not overfit – regardless of the number of trees – due to the existence of a limiting value on the out-of-bag prediction error (Breiman, 2001).

The random model considered here has $m_{\mathrm{try}}$ as its sole hyperparameter. All possible values for $m_{\mathrm{try}} (\{1, 2, ..., 12\})$ were considered in training the model. Training and prediction with the random forest were implemented with the **caret** package (version 6.0-88) (Kuhn, 2008) whereas variable importance for the random forest was analysed with the **randomForest** package (version 4.6-14) (Liaw and Wiener, 2002).

## 4. Bayesian Additive Regression Trees (BART)

Bayesian Additive Regression Trees (BART) (Chipman et al., 2010) is a nonparametric Bayesian *sum-of-trees* model. In contrast to random forest, all trees in a BART ensemble use the full training data instead of a bootstrap sample, and their outputs are summed instead of averaged. Each tree is trained on the residuals left by the previous trees so that each tree models different aspects of the variation in the response variable.

Let $T_{j}$ denote the $j$th binary tree consisting of a set of recursive decision rules based on the predictor values $\boldsymbol{x}$. Each tree in a BART ensemble is a *regression tree*, with terminal nodes assigned real-valued parameters. Let $\mu_{ij}$be the parameter value associated with the $i$th terminal node of tree $T_{j}$. Then $M_{j}= \{\mu_{ij};i = 1,...,b\}$ is the set of parameters associated with tree $T_{j}$, where $g\left( \boldsymbol{\cdot;}T_{j}, M_{j} \right):\boldsymbol{x\mapsto}\mu_{ij}$ is a function which maps a vector of predictor variables $\boldsymbol{x}$ onto the parameter value of its corresponding terminal node $\mu_{ij}$;

| $\Phi^{-1}\left( p_{i} \right)=\sum_{j=1}^{m} g\left( \boldsymbol{x}_{\boldsymbol{i}}\mathbf{;}T_{j}, M_{j} \right) ,$ | (5) |
| --- | --- |

where $\Phi$ is the cumulative distribution function of the standard normal and its inverse $\Phi^{-1}$ is the probit link function.

Given a fixed number of trees $m$, BART has the collection $\{(T_{j},M_{j}),j = 1,...,m\}$ of parameters. BART is a Bayesian model and therefore requires a prior distribution over trees and over parameters of the terminal nodes. The priors act as regularizers, ensuring that the effects of individual trees are controlled (Chipman et al., 2010). Details are below.

### Prior Specification

Under the assumption that individual trees $T_{j}$ are independent and that the parameters $\mu_{i}$associated with the terminal nodes $M_{j}$of each tree are independent and identically distributed (i.i.d.), the prior on the parameters of the BART model is given by

| $p\left( \left( T_{1},M_{1} \right), \ldots, \left( T_{m}, M_{m} \right) \right)=\prod_{j} p(T_{j},M_{j})$ | (6) |
| --- | --- |

| $=\prod_{j} p(M_{j}\vert T_{j})p(T_{j})$ | (7) |
| --- | --- |

| $=\prod_{j} \prod_{i} p(\mu_{ij}\vert T_{j})p(T_{j}) .$ | (8) |
| --- | --- |

Following from the last equation, it suffices to impose priors on the tree structure$, T_{j}$ and on the leaf (terminal node) parameters given the tree structure, $\mu_{ij} |T_{j}$.

Following Chipman, George and McCulloch (2010), the distribution of the prior of the leaf parameters given the tree structure $p(\mu_{ij}|T_{j})$ is assumed to be normal with mean and variance parameters and $\mu_{\mu}$ and $\sigma_{u}^{2}$ respectively, defined such that

$m\mu_{u} - k \sqrt{m}\sigma_{u}= y_{\min}$ and $m\mu_{u}+ k \sqrt{m}\sigma_{u}= y_{\max}$, where $y_{\min}$ and $y_{\max}$ are the minimum and maximum observed values of *Y* respectively and *k* is a constant determining the measure of prior probability on the observed responses. Typically, hyperparameters *m* and *k* are fixed by the investigators. Hyperparameter values considered in this study were $k = 2, 3, 5$ and $m = 50, 200$.

We follow the recommendations of Chipman, George and McCulloch (2010) on the choice of the prior on the tree structure $p(T_{j})$. For the sake of brevity, we omit details on the specification of this prior and refer the interested reader to Chipman, George and McCulloch (2010).

### Posterior Distribution

The BART model uses a Bayesian *backfitting* Markov Chain Monte Carlo (MCMC) algorithm (Hastie and Tibshirani, 2000) for sampling from the posterior distribution $p(\left( T_{1},M_{1} \right),...\left( T_{m},M_{m} \right), Y)$. The algorithm is iterative: the $j$th tree is fitted on the residual resulting from the fit of the sum of all trees excluding the $j$th tree. A brief explanation of the algorithm is now given.

Let $T_{(j)}$ denote the set of all $m - 1$ trees except the $j$th tree and let $M_{(j)}$ denote the set of terminal node parameters for all trees except for the $j$th tree. Posterior samples are obtained using a Metropolis within-Gibbs algorithm as follows (Geman and Geman, 1984; Hastings, 1970). Using a Gibbs sampler (Geman and Geman, 1984), the $jt$h draw $(T_{j},M_{j})$ is obtained, conditional on $T_{(j)}$, $M_{(j)}$. That is,

| $\left( T_{j},M_{j} \right)\vert T_{\left( j \right)}, M_{\left( j \right)}, y, j=1, \ldots, m.$ | (9) |
| --- | --- |

Let $R_{j}$ denote the vector of residuals that result from the fit of all except the $j$th tree. Then,

| $\left( T_{j},M_{j} \right)\vert R_{j}, j=1, \ldots, m$ | (10) |
| --- | --- |

is equivalent to Equation (9), where $R_{j}$ is given as:

| $R_{j} \equiv y- \sum_{k\neq j} g\left( x;T_{k}, M_{k} \right).$ | (11) |
| --- | --- |

The draw from the conditional distribution $\left( T_{j},M_{j} \right)| R_{j}$ is obtained by sampling from $T_{j}|R_{j}$using a Metropolis Hastings algorithm (Chipman et al., 1998; Hastings, 1970), and then from$M_{j}|T_{j}$, which is simply a normal distribution (as seen in Section 2.2.4). The sequence of draws $\{\left( T_{1}^{*},M_{1}^{*} \right),...\left( T_{m}^{*},M_{m}^{*} \right)\}$ thus obtained induces a sequence of sum-of-trees functions $p^{*}(.)$, which converges to the desired target distribution after enough runs (Chipman et al., 2010). The post-burn-in sample is considered an approximate draw from the posterior and may be used in making inferences concerning the true distribution. The posterior mean of was estimated using Markov Chain Monte Carlo after a burn-in of 250 samples.

### Implementation in R

All analyses with the BART model were performed with the **bartMachine** package (version 1.2.6) (Kapelner and Bleich, 2016).

## 5. Multidimensional Monotone BART (mBART)

Chipman et al. (2022) show that in settings where a monotonic relationship exists between predictor(s) and outcome, it is possible to reformulate the BART model to account for such monotonicity thereby enabling better predictions. This reformulation involves the imposition of monotonicity constraints on all BART hyperparameter priors. Using simulations and real data applications, the authors showed that the new model, multidimensional monotone BART (mBART), consistently produced smoother and more precise fits than BART. The current study is an ideal context for the application of mBART since higher antigen reactivity values always indicate increased evidence for *H. pylori* infection, never decreased evidence.

### Prior Specification

As in BART, mBART priors serve to regularize the model fit. Again, as in BART, the independence assumption on trees and the i.i.d. assumption on terminal node parameters apply. The mBART prior is therefore similar to equation (8) except that for mBART, $p(M_{j}|T_{j})$ is constrained to have support for only trees that satisfy some monotonicity conditions (see Chipman et al. for details). The hyperparameters and hyperparameter values considered for $p(M_{j}|T_{j})$ in the BART model are also considered for mBART. We follow the authors’ recommendations on the choice of $p(T_{j})$.

### Posterior Distribution

Realizations from the mBART posterior are obtained by using a slight modification to the Bayesian backfitting MCMC algorithm. The modification involves the use of a localized Metropolis-Hastings algorithm due to the dependence of the $\mu_{i}$’s, which is a consequence of the constraints on $p(M_{j}|T_{j})$. For the sake of brevity, we exclude details on the algorithm. Once approximate draws from the target distribution have been obtained, inference is as is in BART. Posterior samples from the mBART model were drawn after a burn-in of 250 samples.

### Implementation in R

For the implementation of mBART, we utilised the code provided by the authors (available at <https://bitbucket.org/remcc/mbart/src/master/>). We created a custom R interface to the C++ code for the purposes of this study.

# Random forest: predictive performance

The steps involved in obtaining the accuracy-based measure for the importance of a predictor $\boldsymbol{x}_{i}$ are outlined below (Breiman, 2001).

1. For the $j$th tree, calculate the prediction accuracy on the OOB sample.
2. Randomly permute the values of *X_i_* in the OOB sample, while maintaining the order of the other predictors.
3. Calculate the decrease in prediction accuracy of tree *j* on the modified OOB sample as the difference between the accuracies at steps 1 and 2.
4. Repeat steps 1-3 for all trees in the model and average the decrease in accuracy across all trees.

# Cross-validation results

**Table S1: Average out-of-bag (OOB) root mean square error (RMSE) for different hyperparameter combinations for the BART algorithm on the training data.** Scores are displayed for all hyperparameter combinations in order of increasing RMSE. Averages are over validation folds within a 10-fold cross-validation scheme. The hyperparameters $\boldsymbol{m}$and $\boldsymbol{k}$represent the number of trees and the amount of prior information used in the model, respectively. The best hyperparameter combination (that which yielded the smallest OOB RMSE), $\boldsymbol{\{k=2, m=200\}}$, was used for prediction among cases.

| Hyperparameter | |  |
| --- | --- | --- |
| k | m | OOB RMSE |
| 2 | 200 | 0.092 |
| 3 | 200 | 0.094 |
| 3 | 50 | 0.094 |
| 5 | 50 | 0.094 |
| 2 | 50 | 0.098 |
| 5 | 200 | 0.102 |

**Table S2: Average accuracy scores and standard deviations (SDs) for different hyperparameter combinations for the mBART algorithm on the training data**. Averages and SDs are over validation folds within a 10-fold cross-validation scheme. Scores are displayed for all hyperparameter combinations in order of decreasing accuracy. The hyperparameters $m$and $k$represent the number of trees and the amount of prior information used in the model, respectively. The best hyperparameter combination (that which yielded the optimal score across the 10 folds) was used for prediction in the case data. Due to rounding, the accuracy scores of the combinations $\left\{ k=2, m=200 \right\}$and $\{k=3, m=200\}$ appear equal; however, the second set has a higher average score and a lower SD.

| Hyperparameter | |  |
| --- | --- | --- |
| k | m | Accuracy (SD) |
| 3 | 200 | 0.900 (0.05) |
| 2 | 200 | 0.900 (0.05) |
| 5 | 50 | 0.896 (0.05) |
| 3 | 50 | 0.896 (0.04) |
| 5 | 200 | 0.892 (0.05) |
| 2 | 50 | 0.890 (0.04) |

**Table S3: Average accuracy scores and standard deviations (SDs) for different hyperparameter combinations for the random forest algorithm on the training data.** Averages and SDs are over validation folds within a 10-fold cross-validation scheme. Scores are displayed for all possible hyperparameter values in order of decreasing accuracy. The hyperparameter $m_{try}$ represents the number of variables used for node splitting. The best hyperparameter combination (that which yielded the optimal score across the 10 folds), $\{m_{try}=6\}$, was used for prediction in the case data.

| Hyperparameter |  |  |
| --- | --- | --- |
| $\boldsymbol{m}_{\boldsymbol{try}}$ | Accuracy (SD) | |
| 6 | 0.900 (0.03) | |
| 7 | 0.896 (0.03) | |
| 2 | 0.896 (0.04) | |
| 3 | 0.896 (0.04) | |
| 4 | 0.896 (0.04) | |
| 12 | 0.894 (0.02) | |
| 8 | 0.894 (0.03) | |
| 9 | 0.890 (0.03) | |
| 5 | 0.890 (0.04) | |
| 10 | 0.890 (0.04) | |
| 1 | 0.888 (0.04) | |
| 11 | 0.888 (0.04) | |


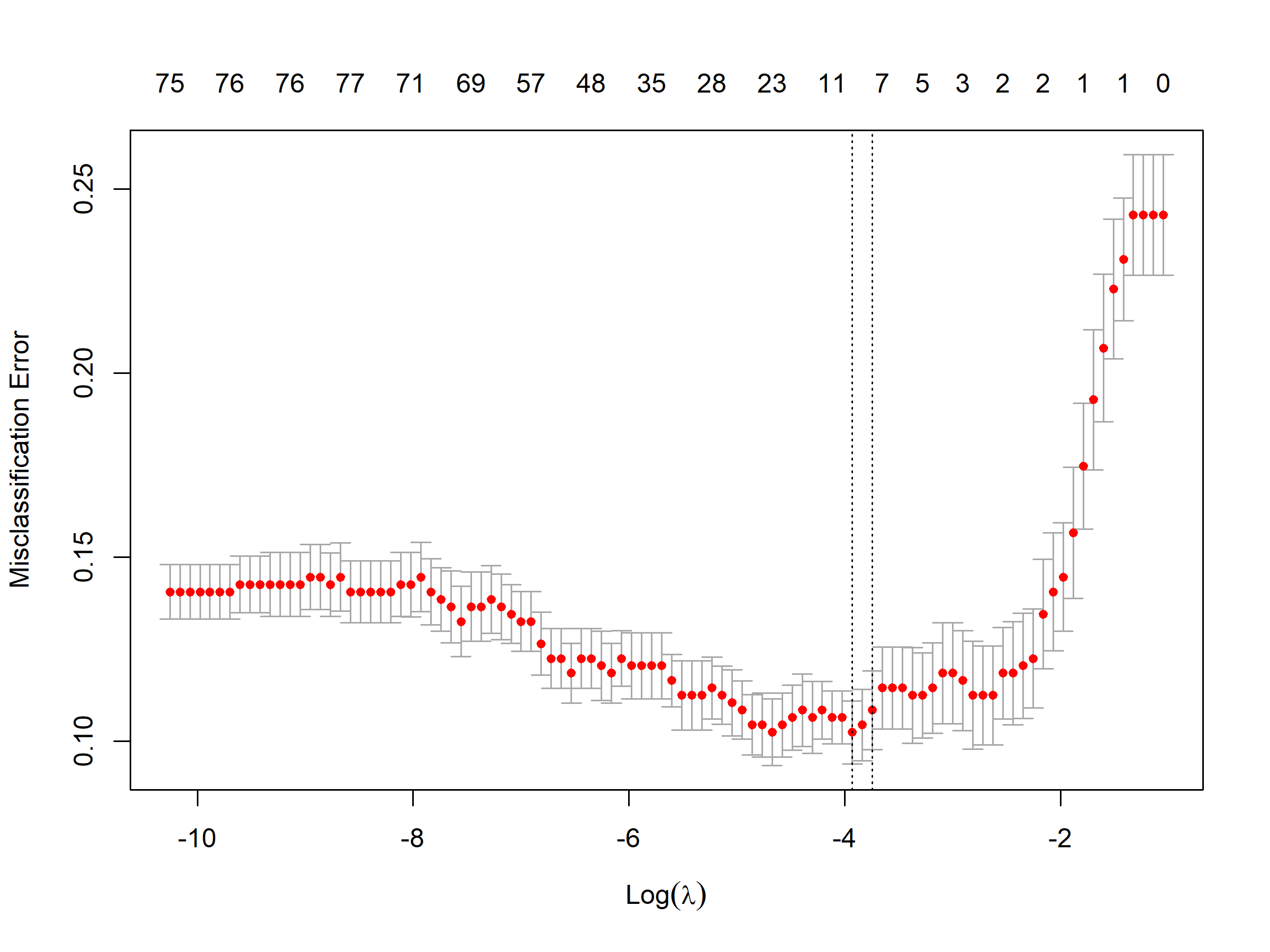


**Figure S1: Misclassification error for different values of** $\boldsymbol{\lambda}$ **in the elastic net (EN) algorithm on the training data within a 10-fold cross-validation (CV) scheme.** Red dots and grey bars represent the average misclassification error and standard deviation of misclassification errors, respectively, across the 10 CV folds for each value of $\lambda$. The dashed vertical lines are plotted at the value of $\lambda$ with the minimum average CV error (first dashed line from the left) and at the largest value of $\lambda$ such that the error is within one standard error of the minimum (second dashed line from the left). The numbers displayed at the top of the plot represent the number of non-zero coefficient estimates at the corresponding values of $\lambda$. The value of $\lambda$ with the minimum average CV error $(\log\left( \lambda\right)= -3.93)$ was used within the EN model for prediction on the case data. The value of $\alpha$ (EN mixing parameter) was fixed at 0.90 to allow more weight towards a lasso penalty ($\alpha$ = 1) while still allowing for a ridge penalty ($\alpha=0)$ to control multicollinearity effects.

# References

Berkson, J., 1944. Application of the logistic function to bio-assay. J. Am. Stat. Assoc. 39, 357–365.

Boehmke, B., Greenwell, B.M., 2019. Hands-on machine learning with R. CRC Press.

Breiman, L., 2001. Random forests. Mach. Learn. 45, 5–32.

Breiman, L., 1996. Bagging predictors. Mach. Learn. 24, 123–140.

Chipman, H.A., George, E.I., McCulloch, R.E., 2010. BART: Bayesian additive regression trees. Ann. Appl. Stat. 4, 266–298.

Chipman, H.A., George, E.I., McCulloch, R.E., 1998. Bayesian CART model search. J. Am. Stat. Assoc. 93, 935–948.

Chipman, H.A., George, E.I., McCulloch, R.E., Shively, T.S., 2022. mBART: Multidimensional Monotone BART. Bayesian Anal. 17, 515–544. https://doi.org/10.1214/21-BA1259

Cox, D.R., 1958. The regression analysis of binary sequences. J. R. Stat. Soc. Ser. B Methodol. 20, 215–232.

Friedman, J., Hastie, T., Tibshirani, R., 2010. Regularization paths for generalized linear models via coordinate descent. J. Stat. Softw. 33, 1–22.

Geman, S., Geman, D., 1984. Stochastic relaxation, Gibbs distributions, and the Bayesian restoration of images. IEEE Trans. Pattern Anal. Mach. Intell. 721–741.

Hastie, T., Tibshirani, R., 2000. Bayesian backfitting (with comments and a rejoinder by the authors). Stat. Sci. 15, 196–223.

Hastie, T., Tibshirani, R., Friedman, J., 2009. The Elements of Statistical Learning : Data Mining, Inference, and Prediction, Second. ed, Springer Series in Statistics. Springer.

Hastings, W.K., 1970. Monte Carlo sampling methods using Markov chains and their applications. Biometrika 57, 97--109.

Hilt, D.E., Seegrist, D.W., 1977. Ridge, a computer program for calculating ridge regression estimates, USDA Forest Service research note NE, 236. Dept. of Agriculture, Forest Service, Northeastern Forest Experiment Station, Upper Darby, PA. https://doi.org/10.5962/bhl.title.68934

Ishwaran, H., 2015. The effect of splitting on random forests. Mach. Learn. 99, 75–118.

Kapelner, A., Bleich, J., 2016. bartMachine: Machine learning with Bayesian additive regression trees. J. Stat. Softw. 70, 1–40. https://doi.org/10.18637/jss.v070.i04

Kuhn, M., 2008. Building predictive models in R using the caret package. J. Stat. Softw. 28, 1–26.

Liaw, A., Wiener, M., 2002. Classification and Regression by randomForest. R News 2, 18–22.

R Core Team, 2021. R: A Language and Environment for Statistical Computing. R Foundation for Statistical Computing, Vienna, Austria.

Tibshirani, R., 1996. Regression shrinkage and selection via the lasso. J. R. Stat. Soc. Ser. B Methodol. 58, 267–288.

Zou, H., Hastie, T., 2005. Regularization and variable selection via the elastic net. J. R. Stat. Soc. Ser. B Methodol. 67, 301–320.
